# Long Term Stabilization of Expanding Aortic Aneurysms by a Short Course of Cyclosporine A through Transforming Growth Factor-Beta Induction

**DOI:** 10.1371/journal.pone.0028903

**Published:** 2011-12-14

**Authors:** Jianping Dai, Stéphanie Michineau, Grégory Franck, Pascal Desgranges, Jean-Pierre Becquemin, Marianne Gervais, Eric Allaire

**Affiliations:** 1 CNRS EAC7054, Centre de Recherches Chirurgicales Dominique Chopin, Faculty of Medicine, Paris-Est University, Créteil, France; 2 Department of Vascular Surgery, Henri Mondor Hospital, Assistance Publique - Hôpitaux de Paris, Créteil, France; King's College London, University of London, United Kingdom

## Abstract

Abdominal aortic aneurysms (AAAs) expand as a consequence of extracellular matrix destruction, and vascular smooth muscle cell (VSMC) depletion. Transforming growth factor (TGF)-beta 1 overexpression stabilizes expanding AAAs in rat. Cyclosporine A (CsA) promotes tissue accumulation and induces TGF -beta1 and, could thereby exert beneficial effects on AAA remodelling and expansion. In this study, we assessed whether a short administration of CsA could durably stabilize AAAs through TGF-beta induction. We showed that CsA induced TGF-beta1 and decreased MMP-9 expression dose-dependently in fragments of human AAAs *in vitro*, and in animal models of AAA *in vivo*. CsA prevented AAA formation at 14 days in the rat elastase (diameter increase: CsA: 131.9±44.2%; vehicle: 225.9±57.0%, P = 0.003) and calcium chloride mouse models (diameters: CsA: 0.72±0.14 mm; vehicle: 1.10±0.11 mm, P = .008), preserved elastic fiber network and VSMC content, and decreased inflammation. A seven day administration of CsA stabilized formed AAAs in rats seven weeks after drug withdrawal (diameter increase: CsA: 14.2±15.1%; vehicle: 45.2±13.7%, P = .017), down-regulated wall inflammation, and increased αSMA-positive cell content. Co-administration of a blocking anti-TGF-beta antibody abrogated CsA impact on inflammation, αSMA-positive cell accumulation and diameter control in expanding AAAs. Our study demonstrates that pharmacological induction of TGF-beta1 by a short course of CsA administration represents a new approach to induce aneurysm stabilization by shifting the degradation/repair balance towards healing.

## Introduction

Abdominal aortic aneurysms (AAAs) result from a protease-driven destruction of the extracellular matrix (ECM) with no significant aortic reconstruction, and account for 15 000 deaths and 33 000 aortic repairs in the USA annually [1]. Current treatments to prevent AAA rupture are hampered by a high post-operative mortality rate for open surgery and a limited durability after endovascular treatment, with comparable four-year all cause mortality rates [2]. These limitations restrain the benefit of screening programs, a strategy of public health interest, since most detected patients carry small AAAs with currently no alternative to surveillance [1].

Accordingly, research has focused on pharmacological approaches to stop expansion of AAAs [3], [4], [5] by a regimen of continuous drug administration to suspend the aortic destructive process. We thought it of interest to develop a pharmacologic approach by which a short course of drug administration would allow for a long term control of AAA diameter beyond treatment interruption. Such a curative, rather than suspensive, treatment would open up the possibility to use potent modulators of the balance between aortic destruction and repair with limited side effects. Using an endovascular gene therapy approach, we have shown previously that a transient overexpression of TGF-beta1 resulted in a long term stabilization of diameter in formed, expanding AAAs [6]. As a mechanistic explanation for the durability of this effect, we documented induction of endogenous TGF-beta1 gene relaying the rapidly neutralized transgene expression, and the correction of VSMC depletion [6], a characteristic of AAAs [7]. This work and other data from our laboratory [8] have pointed to the potential of TGF-beta1 induction to reprogram the diseased aortic wall at a cellular level, thereby restoring the capacity of the repaired aneurysmal aorta to withstand hemodynamic stress without further dilatation.

Cyclosporine A (CsA) is an immunosuppressive drug of the calcineurin inhibitor family which induces TGF-beta1 gene transcription and activates latent TGF-beta [9]. Numerous side effects of chronic administration of CsA in humans, including nephropathy [10] and gingival hypertrophy [11], demonstrate its ability to promote tissue accumulation. Notably, ECM accumulation after CsA administration relies on TGF-beta activity [10]. Here, we tested the hypothesis that a short course of CsA administration could shift the aneurysmal destruction/reconstruction balance, and represent a new pharmacological strategy to restore AAA wall integrity and stability. We demonstrate that CsA induced TGF-beta1 in fragments of human AAAs *in vitro* and in two animal models of AAA, and prevented AAA formation in the elastase and the calcium chloride (CaCl_2_) models. Concordant with our hypothesis, a short administration of CsA durably stabilized the diameter of formed AAAs while increasing their VSMC content. Moreover, the co-administration of an anti-TGF-beta1 blocking antibody abrogated CsA impact on AAA diameter control and VSMC content. We propose that induction of TGF-beta1 by a short course of CsA administration represents a new pharmacological approach to durably control aneurysm diameter.

## Results

### CsA in human AAA explants

**Figure 1:**
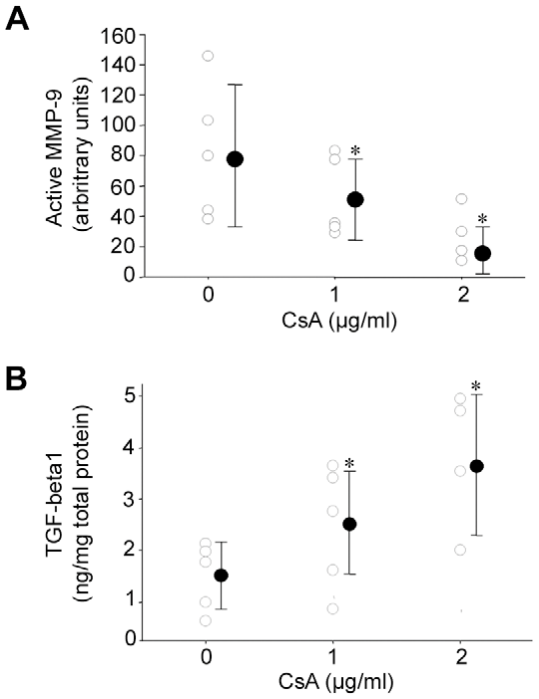
CsA modulates TGF-beta1 and MMP-9 secretion from human AAAs *in vitro*. Quantification of TGF-beta1 and MMP-9 in 24 h-conditioned media obtained from human AAA explants incubated or not with CsA. Open circles represent individual protein levels and closed circles represent means±SD. *P<.05 vs untreated-CsA group.

Because TGF-beta and MMP-9 are representative of the reconstruction/destruction process occurring in the aneurismal wall [12], we evaluated the impact of CsA on their secretion in 24 h-conditioned medium from explants from five different human AAAs. Addition of CsA on AAA explants (within the range of CsA concentrations observed in total blood under current clinical use) dose-dependently increased TGF-beta1 and decreased MMP-9 protein secretions (Figure 1).

### CsA and AAA formation in rodents

#### CsA prevents AAA development

**Figure 2:**
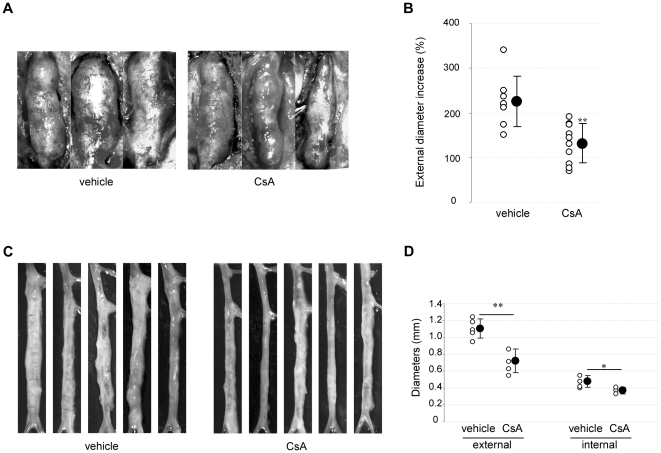
CsA prevents AAA development in the rat elastase and the mouse CaCl_2_ models. A. Macroscopic pictures of representative rat AAAs 14 days after elastase perfusion. B. External diameter increase at 14 days as a percentage of AAA diameters just after elastase perfusion. C. Macroscopic pictures of mouse AAAs at 14 day. D. Mouse external and internal aortic diameter quantification. Open circles represent individual values from vehicle- and CsA-treated animals and closed circles represent means±SD. *P<.05, **P<0.01 *vs* vehicle.

The effect of CsA was evaluated in the rat elastase and mouse CaCl_2_ models. Fourteen days after elastase perfusion, the increase in aortic diameter was smaller in CsA- than in vehicle-treated rats (external diameter increase: 131.9±44.2 *vs* 225.9±57.0%, respectively, P = 0.0034) (Figure 2A and 2B). Similarly, 14 days after CaCl_2_ application, diameters were smaller in CsA- than in vehicle-treated mice (external diameter: 0.72±0.14 *vs* 1.10±0.11 mm, respectively, P = .008; internal diameter: 0.37±0.04 *vs* 0.48±0.07 mm, respectively, P = .028) (Figure 2C and 2D). These results demonstrate that CsA prevents AAA formation in two rodent models.

#### CsA prevents VSMC loss and elastin destruction

**Figure 3:**
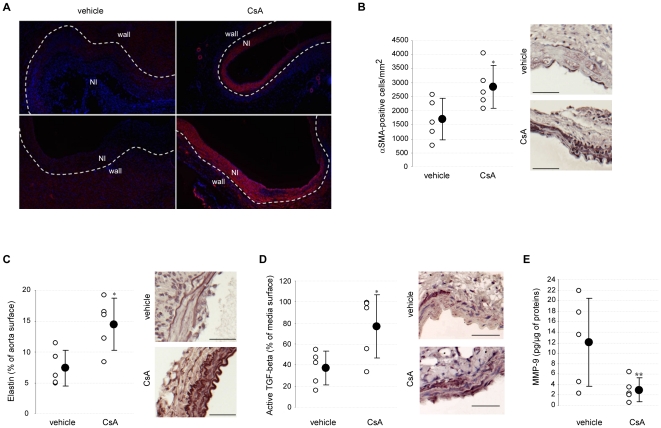
CsA preserves VSMC and elastin content and modulates TGF-beta1 and MMP-9 expression in mouse AAAs. A. Representative anti-αSMA immunostaining performed on AAA cross sections from vehicle- or CsA-treated rats, 14 days after elastase perfusion (red: αSMA staining; blue : nuclei). B, C. Representative anti-αSMA (B) or elastin fibers (C) staining (*right*) and computer-assisted quantification (*left*) performed on AAA cross sections from vehicle- or CsA-treated mice at 14 days. D. Representative anti-active TGF-beta1 staining *(right)* and computer-assisted quantification *(left)* performed on AAA cross sections from vehicle- and CsA-treated mice at 14 days. E. ELISA quantification of MMP-9 on AAA extracts from vehicle- and CsA-treated mice. Results are reported to the total protein level. Open circles represent individual values from vehicle- and CsA-treated mice and closed circles represent means±SD. *P<.05, **P<.01 *vs* vehicle. NI : neointima; ILT: intraluminal thrombus. Scale bars : 50 µm.

Prevention of AAA formation by CsA was accompanied by an increased density of αSMA-positive cells in the neointima in elastase-perfused aortas (Figure 3A) and by a higher number of αSMA-positive cells in the media in mice (αSMA-positive cells per mm^2^: 2856±765 *vs* 1697±732 in CsA- and vehicle-treated mice, respectively, P = .047) (Figure 3B). Morever, in the CaCl_2_ model, CsA preserved the aortic medial elastic network structure and density (elastic fiber surface: 14.5±4.2 and 7.4±2.9% of the aorta surface in CsA- and vehicle-treated mice, respectively, P = .028) (Figure 3C). Altogether, these results demonstrate that CsA prevents aortic wall destruction.

#### CsA increases TGF-beta1 and decreases MMP-9 expression

In mice, the prevention of AAA formation by CsA was paralleled by a significant increase in anti-TGF-beta1 immunostaining localized in the medial layer (active TGF-beta staining: 77±30 *vs* 37±16% of the media surface in CsA- and vehicle-treated mice, respectively, P = .047) (Figure 3D). CsA also reduced total MMP-9 content in mouse AAA extracts (total MMP-9 level: 3.01±2.22 *vs* 12.08±8.41 pg/µg of total proteins in CsA- and vehicle-treated mice, respectively, P = .008) (Figure 3E).

### Pharmacologic induction of stabilization of expanding AAAs by short-term CsA administration in rats

#### A short treatment with CsA induces long-term stabilization of already-formed AAAs in rats

**Figure 4:**
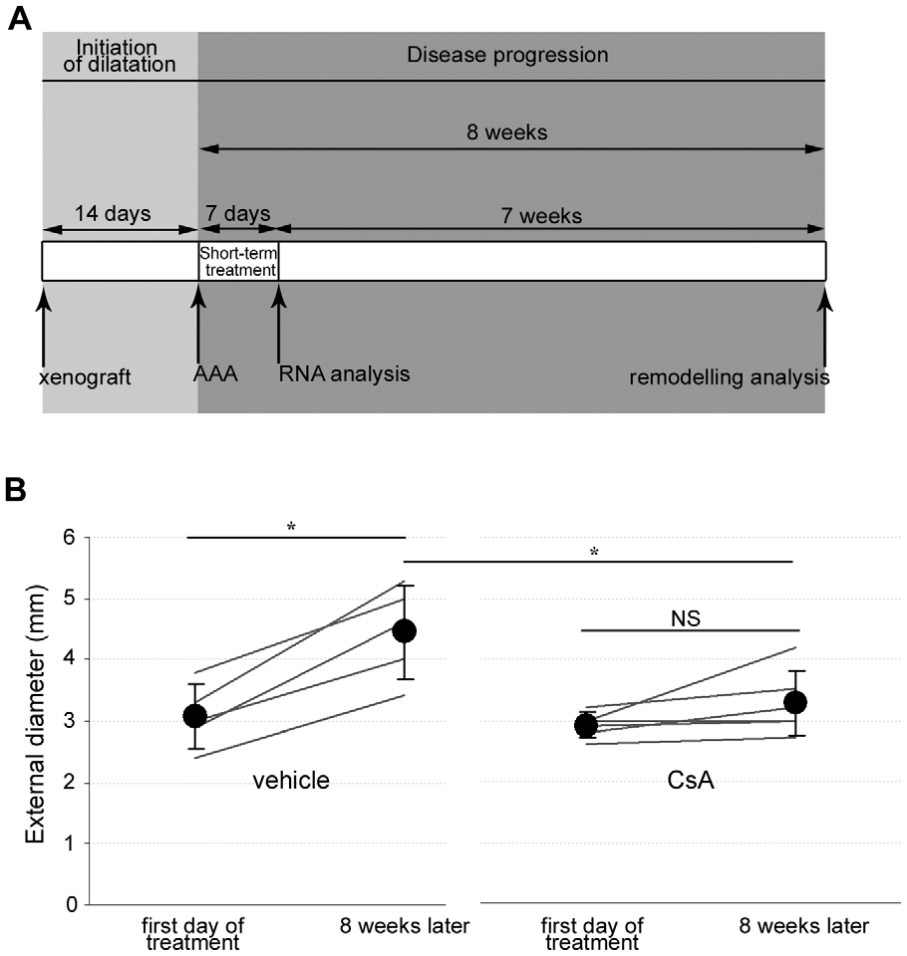
Short CsA treatment induces durable stabilization of AAAs in rats. A. Experimental design: rats with already formed, expanding AAAs were treated for 7 days with vehicle or CsA. The percentage of diameter increase seven weeks after treatment withdrawal was calculated with reference to AAA diameter at CsA introduction. Analysis of mRNA contents was performed at the end of the 7-day treatment, with the assumption that biological changes at this delay provide early mechanistic explanations for the impact of CsA on the remodeling observed at 8 weeks. B. Impact of the 7-day CsA administration on AAA expansion 7 weeks after treatment withdrawal. Open circles represent individual external aortic diameters and closed circles represent means±SD. *P<.05.

We then addressed whether a short-term administration of CsA induces stabilization of expanding AAAs durably after treatment interruption. For this purpose, we used the xenograft model of AAA that mimics important evolutive and structural features of human atherosclerotic AAAs, such as constant expansion, inflammatory and proteolytic burden, and intraluminal thrombus [13], [14]. CsA was administrated subcutaneously for seven days on already-formed AAA. AAA remodelling was assessed 7 weeks after CsA treatment interruption (Figure 4A).

As expected, the external diameter of abdominal aortas had significantly increased 14 days after xenograft implantation and was not different between CsA- and vehicle-treated rats at the time of CsA treatment initiation (2.9±0.2 *vs* 3.3±0.5 mm, respectively, NS) (Figure 4B). Whereas AAA diameter continued to expand significantly in vehicle-treated rats (P<0.05), short CsA treatment suspended AAA expansion up to 7 weeks after drug withdrawal (diameter increase at 8 weeks: 14.2±15.1 and 45.2±13.7% in CsA- and vehicle-treated rats, respectively, P = .017) (Figure 4B).

#### Induction of AAA stabilization by CsA parallels with increased VSMC aortic content, decreased aortic inflammation, a shift of MMP-dependent proteolytic balance and an upregulation of TGF-beta1 expression

**Figure 5:**
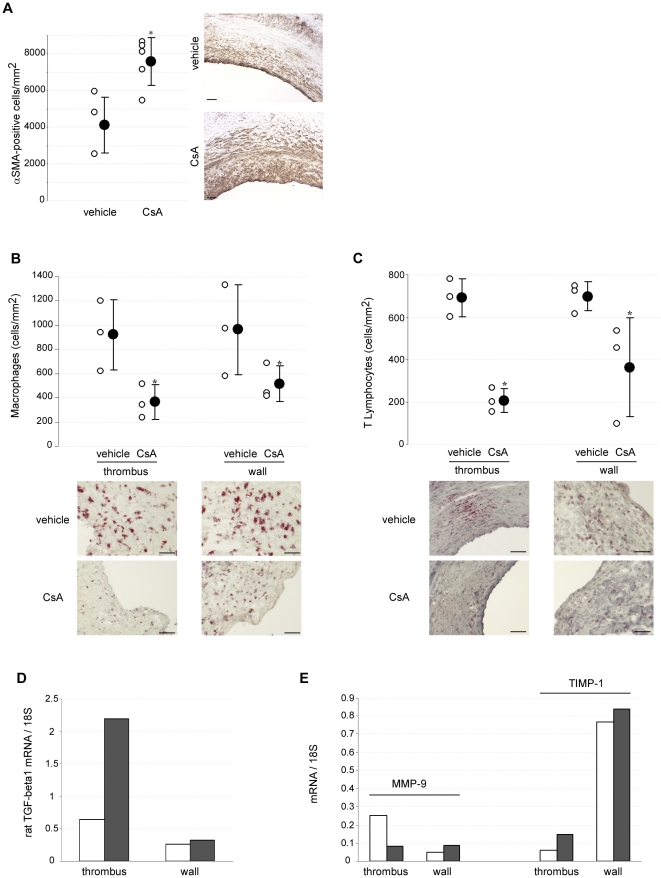
CsA modulates VSCM density, inflammation and TGF-beta1, MMP-9 and TIMP-1 expression in already-formed AAAs in rats. A, B and C. Representative immunostaining (*lower panel*) and quantification (*upper panel*) of αSMA-positive cells (A), macrophages (B) and T-lymphocytes (C) performed on rat AAA cross sections, 7 weeks after CsA withdrawal. Open circles represent individual cell densities and closed circles represent means±SD. D and E. RT-PCR semi-quantification of mRNAs encoding TGF-beta1, MMP-9 and TIMP-1, expressed as ratios to 18 s mRNAs after a 7-day treatment. White columns: vehicle (n = 5); black columns: CsA (n = 6); *P<.05 *vs* vehicle. Scale bars: 50 µm.

Stabilization of expanding AAA by CsA was associated with a higher number of αSMA-positive cells in the intima/thrombus, 7 weeks after drug withdrawal (αSMA-positive cells per mm^2^: 7583±1313 *vs* 4137±1513 in CsA - and vehicle-treated rats, respectively, P = .017) (Figure 5A). Furthermore, CsA decreased monocyte-macrophage density in formed AAAs (thrombus: 367±142 *vs* 923±290; media/adventitia: 517±149 *vs* 962±375 ED1-positive cells per mm^2^ in CsA- and vehicle-treated groups, respectively, P = .049) (Figure 5B), as well as T lymphocyte infiltration (thrombus: 209±57 *vs* 694±89; media/adventitia: 364±232 *vs* 781±181 R73-positive cells per mm^2^ in CsA- and vehicle-treated groups, respectively, P = .049) (Figure 5C). At the transcriptional level, CsA led to a 4-fold increase in TGF-beta1 mRNA content (Figure 5D), decreased MMP-9 and increased TIMP-1 mRNA content in the thrombus (Figure 5E).

These results indicate that a short treatment with CsA increases VSMC content in the aortic wall, induces TGF-beta expression, decreases AAA inflammation and shifts the MMP-dependant proteolytic balance towards inhibition.

### induction of AAA stabilization by CsA is mediated by TGF-beta

To assess the role of TGF-beta activity in CsA-induced AAA stabilization, a TGF-beta-neutralizing antibody was administrated to rats with expanding AAAs treated by CsA.

**Figure 6:**
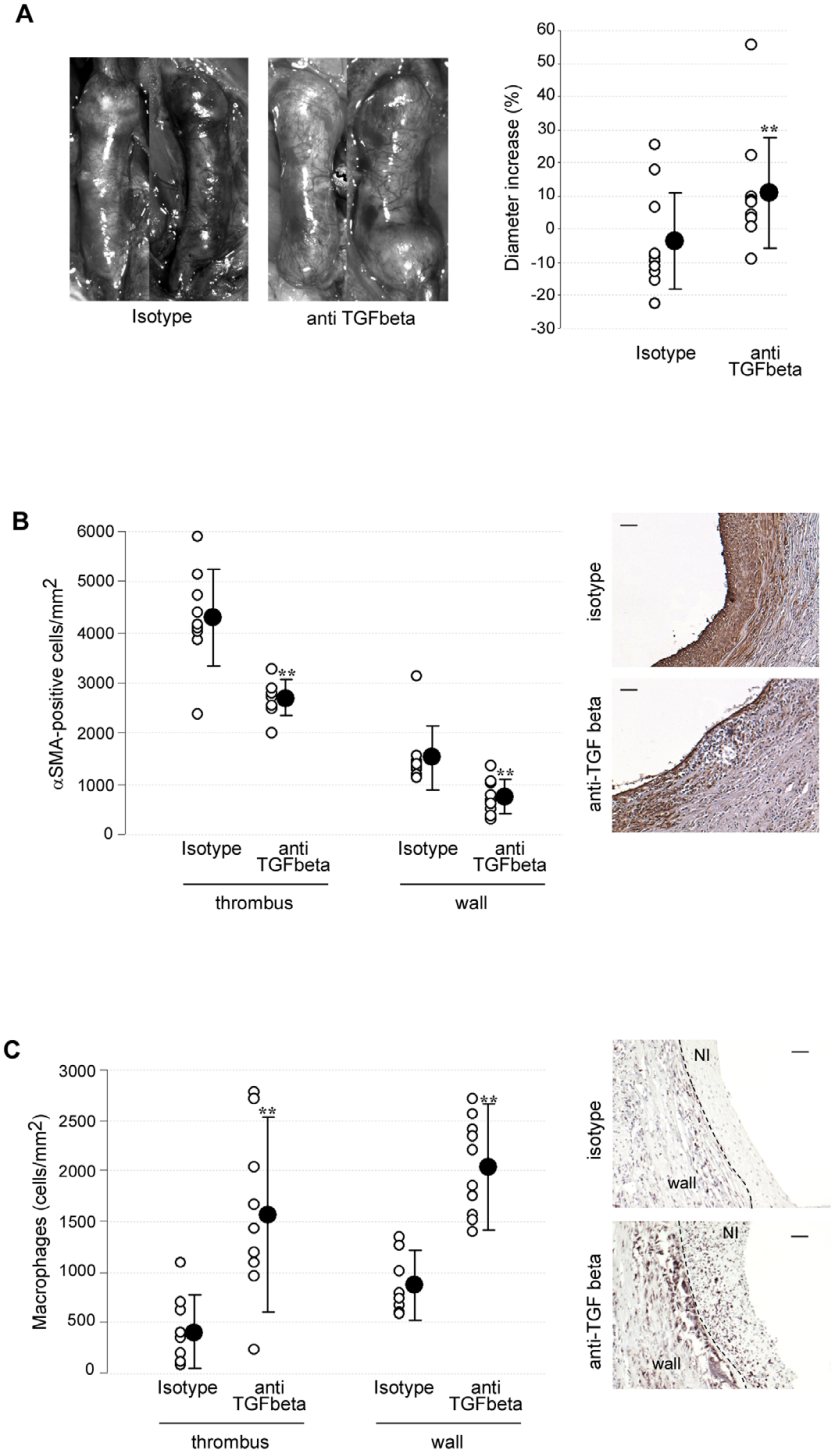
CsA induction of AAA stabilization is mediated by TGF-beta. A. Effect of the administration of a blocking antibody against active TGF-beta on the stabilizing effect of CsA on aortic diameter of expanding AAAs in rats. B and C. Impact of anti-TGF-beta blocking antibody on αSMA-positive cell density (A) and monocyte/macrophage infiltration (C) in rat AAAs treated with CsA. Open circles represent individual values and closed circles represent means±SD. **P<.01 *vs* isotype antibody.

The stabilizing effect of CsA was conserved in control rats but was abrogated in rats injected with the neutralizing antibody (aortic diameter increase at 4 weeks: 10.9±16.7 and −3.6±14.5% in neutralizing- and isotype control antibody-treated rats, respectively, P = .049) (Figure 6A). In contrast, in animals treated by vehicle instead of CsA, administration of the neutralizing antibody against TGF-beta had no effect on AAA diameter variation (aortic diameter increase at 4 weeks: 10.6±3.7 and 11.7±7.0% in neutralizing- and isotype control antibody-treated rats, respectively, P = NS), demonstrating that the protective effect of CsA on AAA expansion is mediated by TGF-beta.

Inhibition of CsA effects by TGF-beta1 neutralizing antibody led to a significant decrease in αSMA-positive cell density (media/adventitia: 745±346 *vs* 1520±624; thrombus: 2700±362 *vs* 4300±965 αSMA-positive cells per mm^2^ in neutralizing- and isotype antibody-treated groups, respectively, P = .001 and .005), 3 weeks after CsA retrieval (Figure 6B). Besides, TGF-beta neutralizing antibody injection was paralleled by an increase in monocyte-macrophages infiltrating AAAs (media/adventitia: 2031±471 *vs* 871±279; thrombus: 1569±832 *vs* 412±341 ED1-positive cells per mm^2^ in neutralizing- and isotype control antibody-treated groups, respectively, P<.01) (Figure 6C).

These results demonstrate that long-lasting AAA stabilization by a pulsed treatment with CsA is mediated by TGF-beta that maintains aortic VSMC density and exerts inhibitory effects on inflammatory cell recruitment.

## Discussion

This is the first study to demonstrate that a short course of drug administration can stabilize the diameter of expanding AAAs durably after treatment withdrawal, by inducing TGF-beta1. CsA induces TGF-beta1 in three models of AAA, including in human AAA wall *in vitro*. CsA administration prevents AAA formation in mice and rats and a seven day CsA administration durably stabilizes formed, expanding AAAs in rats. Co-administration of an anti-TGF-beta blocking antibody suppresses the inhibitory action of CsA on ECM destruction, VSMC accumulation and AAA expansion.

The rationale for using CsA in our study stems from its ability to induce TGF-beta1 [10], a growth factor and cytokine with pleïotropic activities. CsA triggers TGF-beta1 expression in VSMCs [15] and fibroblasts [16] *in vitro*, and *in vivo* in kidney [17], gingival [11] and arterioles [18]. Here, we document that CsA induces TGF-beta1 in two animal models of AAA and in human samples of atherosclerotic AAAs.

In parallel with the enhanced TGF-beta signaling, our results show that CsA administration prevents aneurysmal degeneration in two rodent models of AAA, as previously evoked using a cocktail of immunosuppressive drugs including CsA in the rat elastase model [19]. Of interest, CsA treatment in our study stabilized the diameter of formed, expanding AAAs in rats. Importantly, co-administration of an anti-TGF-beta blocking antibody suppressed the inhibitory action of CsA on AAA expansion, showing that CsA exerted its stabilizing effect through TGF-beta induction. At first sight this may appear contradictory with data showing that exacerbated TGF-beta1 signaling in monogenic defects is associated with aneurysms and dissections of the ascending aorta in human and mice [12], [20]. However, lesions of the ascending aorta differ from atherosclerotic AAAs, with respect to VSMC and ECM content, inflammation and thrombus accumulation. In addition, VSMCs respond differently to TGF-beta 1 according to their embryologic origin which differs along the adult vascular tree [20]. Nevertheless, our data are in agreement with our previous demonstration that TGF-beta1 overexpression by endovascular gene therapy stabilizes expanding AAAs [6] and with a recent confirmation that TFG-beta 1 controls aortic enlargement in a mouse model [21].

Transmural inflammatory cell infiltration is an important feature of AAAs. Infiltrating leukocytes are thought to be a major source of proteases directed against aortic ECM in human and experimental AAAs [12], [22]. CsA has been shown to decrease MMP-9 expression in infarcted myocardium [23] and in rat glomerular mesengial cells [24]. Moreover, CsA decreases MMP-2 activation in VSMCs, another cellular source of proteases in AAAs [25]. Here, we confirm in the three models of AAAs the ability of CsA to favorably shift the proteolytic balance. CsA dose-dependently decreases MMP-9 release by human AAA explants, decreases aortic MMP-9 expression in the two animal models and increased aortic TIMP-1 expression in rats. Down-regulation of inflammation and proteolysis is likely to be one of the mechanisms by which CsA prevents or stabilizes AAA expansion, as further suggested by the preservation of elastic fiber network in the CaCl_2_ model. Furthermore, in the aneurysmal wall and/or thrombus in rats, we show that CsA administration decreases infiltration by macrophages and T lymphocytes, which have been shown to increase the proteolytic burden in AAAs [26]. In a same way, CsA has been shown to decrease inflammation in mechanically injured arteries [27]. The mechanisms by which CsA may decrease inflammation are multiple and controversial. Here, using a blocking strategy, we demonstrate that TGF-beta activity is required for CsA to down-regulate inflammation in formed AAAs.

VSMC loss is another histopathological important feature of human AAAs [12]. Conversely, addition of VSMCs stabilizes the diameter of expanding AAAs [28]. Here, we show that the preventive and curative effects of CsA on AAAs are paralleled with aortic wall VSMC accumulation. Those results are compatible with the fact that one hallmark of CsA-induced vasculopathy in organ transplantation is the inappropriate accumulation of VSMCs [29]. Most importantly, in our study, the blocking antibody strategy credit the notion that CsA-induced TGF-beta1 preserves and restores VSMC content in AAAs, while controlling inflammation and proteolysis. Many data suggest that TGF-beta 1 is important in strengthening the vasculature, a concept supported by our findings. TGF-beta1 promotes VSMC accumulation in normal and atherosclerotic arteries [30], [31]. Besides, in Angiotensin II-infused mice, exacerbation of aortic dilatation and rupture upon neutralization of TGF-beta activity is paralleled by a decrease in aortic VSMC content [21].

Of interest, our study further demonstrates that a seven day treatment with CsA is sufficient to stabilize the diameter of expanding AAAs seven weeks after treatment withdrawal. This result supports the view that a short pulse of CsA administration leads to a long-lasting reprogrammation of the aortic wall towards healing, possibly through TGF-beta induction loop.

### Clinical implications

Induction of healing represents a strategy to stabilize AAAs and improve durability of current prosthetic endovascular approaches. For this purpose, our laboratory and other groups have promoted cell [28], [32], [33] or gene [6] therapy, two approaches hampered by technical difficulties and potential high cost for a frequent disease. From a pragmatic standpoint, correcting VSMC depletion in AAAs using a pharmacological approach would obviate these limitations in a clinical setting. Our study provide a model of pharmacological induction of cellular repair of AAAs by turning-on a self-promoting cytokine with pleïotropic activities, as an alternative to gene or cell therapy. Our data opens up the possibility of using a short drug administration to control durably AAA expansion in patients.

## Methods

The study on human aortic samples was approved by a local ethic committee (Comité de Protection des Personnes, CPP Mondor approval N°09-017). An informed written consent was obtained from all participants in the study. All experimental procedures were conducted in conformity with European (86/609/EEC) rules for animal care and have been approved by the French Veterinary Department (approval N°94-226).

### Models of aneurysm

#### Human AAA explants

Fragments from five asymptomatic human atherosclerotic AAAs (>55 mm) collected during elective surgery in the Henri Mondor Hospital, were cultured in serum-free medium with or without 1 or 2 µg/ml CsA (Sandoz, Rueil-Malmaison, France) at 37°C. After 24 hours, explant-conditioned media were snap-frozen.

#### The CaCl_2_ model in mice

AAAs were generated in 8 week-old C57Bl/6 male mice (Charles River Laboratories) by periaortic application of CaCl_2_ [34]. Mice received CsA (50 mg/kg daily, n = 5) or vehicle (n = 5) intraperitoneously, starting two days before CaCl_2_ application, until harvest. At day 14 after CaCl_2_ application, mice were anesthetized. After intracardiac perfusion of saline, infrarenal aortas were cleared of surrounding tissue, photographed *in situ* [34] and cut in two pieces, one being fixed in 4% paraformaldehyde and decalcified overnight in 0.5 M EDTA before paraffin embedding, the other snap-frozen in liquid nitrogen and kept at −80°C.

#### The elastase model in rats

AAA were generated in 250 g male Wistar rats (Charles River Laboratories) by infusing five units of pancreatic porcine elastase (Sigma, E-1250 lot No. 083K7655), for 1 hour in the aortic lumen, as previously described [35]. Rats received CsA (5 mg/kg/day, n = 10) or vehicle (n = 7) subcutaneously for 14 days, starting immediately after elastase infusion and wound closure. The subrenal aorta was photographed *in situ* under beating heart immediately after elastase infusion and 14 days later. AAAs were explanted, fixed in 70% ethanol and embedded in paraffin.

#### The xenograft model in rats

AAAs were generated in 250 g male Fischer 344 rats (Charles River Laboratories) by implanting an aortic xenograft [36]. Rats with developed AAAs (e.g. 14 days after xenograft implantation) received subcutaneously the vehicle (control group, n = 5) or CsA 5 mg/kg/day (n = 6) for seven days. The subrenal aorta was photographed *in situ* under beating heart, at the time of initiation of CsA treatment, and eight weeks later, e.g. seven weeks after treatment withdrawal, before euthanasia. AAAs were fixed in 70% ethanol and embedded in paraffin. AAAs from six others rats (three in each group) were harvested at day seven after CsA treatment initiation. Thrombi and aneurysmal wall were separately snap-frozen and kept at −80°C. To investigate the role of TGF-beta in CsA-induced effects, rats with formed AAAs, treated with CsA, received an intraperitoneal injection of 0.5 mg/kg of a pan-specific anti-TGF-beta monoclonal antibody (clone 1D11, R&D Systems, Lille, France) three times/week for two weeks, starting on the first day of CsA treatment. This antibody has neutralizing properties against TGF-beta1, 2 and 3 bioactivity [37]. As a control, we used the isotype monoclonal antibody (clone 11711, R&D Systems). AAAs were harvested three weeks after CsA withdrawal.

### Histology, immuno-histochemistry and -fluorescence

Five µm thick paraffin-embedded cross sections were used for mice and rat AAAs.

For histological analysis, sections were stained with orcein for visualization of elastic fibers. For immuno-histochemical analysis, sections were incubated with the following antibodies: mouse monoclonal anti-alpha smooth muscle actin (αSMA, clone 1A4, Sigma-Aldrich, Lyon, France), mouse anti-rat TCR alpha/beta (Vector Laboratories, Abcys, Paris, France), mouse anti-rat monocyte-macrophage (clone ED1, Serotec, Düsseldorf, Germany), and rabbit anti-active TGF-beta (Promega, Charbonnele, France). When mouse primary antibody was used on mouse sections, the Vector M.O.M kit was used, according to the manufacturer's instructions. After incubation with a biotin-conjugated anti-species antibody (Vector Laboratories), immunostaining was amplified using peroxydase-conjugated streptavidin complexes (Vector Laboratories) and peroxydase was detected using VIP (Vector Laboratories) or Fast Red substrate System (Dako, Trappes, France). Sections were counterstained with hematoxylin, mounted in Eukitt and examined with a bright field microscope (Zeiss, France).

For immuno-fluorescence study, sections were incubated with a cyanine 3-conjugated anti-αSMA (clone 1A4, Sigma). Nuclei were stained with DAPI and sections were mounted in Mowiol. Fluorescence was examined with a fluorescence microscope (AxioImager D1, Zeiss) in sequential scanning mode for double detection of cyanine 3 and DAPI. Mosaic images were obtained with a 20× objective lens.

### Computer-assisted morphometric analysis

*In situ* macroscopic and microscopic images were digitally captured using the Axiovision 4.8 Software (Zeiss). Customized programs were used to quantify the remodeling of the vessels, the elastic fiber content, the inflammatory infiltrate, the VSMC density and the TGF-beta expression level. The observer (SM) was blinded to treatment allocation.

#### Remodeling of the vessels

External diameters were measured from *in situ* images of infrarenal aortas using Axiovision. Measurements of internal diameter in mice were performed on cross-sections. The internal diameter corresponds to the diameter of the equivalent circle having the same perimeter as the aortic lumen.

#### Elastic fiber content

The orcein-stained surface was quantified on histological cross-sections using AxioVision. Briefly, the software allows for the selection and subsequent quantification of pixel intensities in a chosen color spectrum (red-brown corresponding to orcein staining). In order to minimize variations between histological preparations, all microscopic slides were stained simultaneously in the same orcein bath, the same range of pixel intensity was used for all the quantifications, and the observer was blinded for the treatment group. Results were expressed as a percentage of total aortic surface.

#### Aortic Inflammatory Infiltrate, medial VSMC density and active TGF-beta expression

Macrophages, T lymphocytes and VSMCs in aortic sections were quantified after immunostaining (see above) and expressed as the number of cells per mm^2^ of total aortic surface. The surface of active TGF-beta1 in aortic sections was quantified after immunostaining and expressed as the percentage of the media surface.

### Elisa quantification of TGF-beta1 in human explant and MMP-9 in mouse models

ELISA kits (R&D Systems) were used to quantify TGF-beta1 in human aortic conditioned media or MMP-9 in mouse aortic extracts. Proteins from mouse aortas were prepared by homogenizing tissues with a potter in a ice-cold extraction buffer (1% Nonidet P-40 in 50 mM Tris-HCl, pH 7.4, containing 120 mM NaCl, 1 mM EDTA, 50 mM NaF, and protease inhibitors (Sigma-Aldrich)) after aorta pulverization with a MultiSample Bio-Pulverizer (Biospec, Bartlesville, USA). Results were normalized to total protein content, determined by BCA quantification.

### Quantification of MMP-9 by zymography in human explant

MMP-9 activity was evaluated by gelatin zymography [28]. Briefly, 5 µg of protein from human AAA conditioned medium were subjected to a 10% SDS-PAGE containing 0.1% gelatin. After electrophoresis, gels were washed in 2.5% Triton X-100 for 30 minutes, further incubated for 24 hours at room temperature in 50 mmol/l Tris, pH 8.2 containing10 mmol/l CaCl_2_, and finally stained with 0.008% Coomassie brilliant blue (Sigma Chemical Co). Quantitative analysis of MMP-9 activity was performed using QuantityOne software (Bio-Rad, Hercules, CA).

### Analysis of mRNA levels by comparative RT-PCR

Because of the small amount of material available for separate analysis of wall and thrombus in the rat model, tissues from 3 rats were pooled by group and layer. Total RNAs from pooled luminal thrombus or aneurysmal wall (media/adventitia) were extracted with Trizol reagent (Invitrogen, Cergy Pontoise, France). Reverse transcription (RT) was performed with random primers (Roche, Meylan, France), M-MLV reverse transcriptase, dNTP, and ribonuclease inhibitor (Eurobio, Les Ulis, France). Semiquantitative PCR reactions were performed in presence of primers for the domestic 18S gene RNA (QuantumRNA 18S Internal Standards Kit, Ambion, Courtaboeuf, France) and specific rat primers: Tissue Inhibitor of MetalloProteinase-1 (TIMP-1) (*forward*: 5′-CCCCAGAAATCAACGAGAGACCA-3′; *reverse*: 5′-ACACCCCACAGCCAGCACTAT-3′), Matrix MetalloProteinase 9 (MMP-9) (*forward*: 5′-CTGCGTATTTCCATTCATCTT-3′; *reverse*: 5′-ATGCCTTTTATGTCGTCTTCA-3′) and TGFbeta-1 (*forward*: 5′-CGGACTACTACGCCAAAGAA-3′; *reverse*: 5′-TCAAAAGACAGCCACTCAGG-3′).

PCR products were run on agarose gels with ethidium bromide. Band intensity of amplified sequences were visualized under UV light by a video camera and quantified with Gel Analyst software. Results were expressed as the ratio between gene of interest and 18S signals.

### Statistics

Quantitative data are expressed as means±SD. All statistical tests were non parametric. Comparisons between more than two groups were done with the Kruskall-Wallis test, and if significant (P<0.05) followed by two-by-two comparisons. Mann-Whitney test was used for all two-by-two comparisons. P<.05 was considered as statistically significant.
